# Enhancement of aragonite mineralization with a chelating agent for CO_2_ storage and utilization at low to moderate temperatures

**DOI:** 10.1038/s41598-021-93550-9

**Published:** 2021-07-06

**Authors:** Jiajie Wang, Noriaki Watanabe, Kosuke Inomoto, Masanobu Kamitakahara, Kengo Nakamura, Takeshi Komai, Noriyoshi Tsuchiya

**Affiliations:** Graduate School of Environmental Studies, Tohoku University, Aramaki, Aoba-ku, Sendai, 9808579 Japan

**Keywords:** Climate sciences, Environmental sciences, Solid Earth sciences, Materials science

## Abstract

Among the CaCO_3_ polymorphs, aragonite demonstrates a better performance as a filler material in the paper and plastic industries. Despite being ideal from the environmental protection perspective, the production of aragonite particles via CO_2_ mineralization of rocks is hindered by the difficulty in achieving high production efficiencies and purities, which, however, can be mitigated by exploiting the potential ability of chelating agents on metal ions extraction and carbonation controlling. Herein, chelating agent *N,N*-dicarboxymethyl glutamic acid (GLDA) was used to enhance the extraction of Ca from calcium silicate and facilitate the production of aragonite particles during the subsequent Ca carbonation. CO_2_ mineralization was promoted in the presence of 0.01–0.1 M GLDA at ≤ 80 °C, with the maximal CaCO_3_ production efficiency reached 308 g/kg of calcium silicate in 60 min using 0.03 M GLDA, which is 15.5 times higher than that without GLDA. In addition, GLDA showed excellent effects on promoting aragonite precipitation, e.g., the content of aragonite was only 5.1% in the absence of GLDA at 50 °C, whereas highly pure (> 90%, increased by a factor of 18) and morphologically uniform aragonite was obtained using ≥ 0.05 M GLDA under identical conditions. Aragonite particle morphologies could also be controlled by varying the GLDA concentration and carbonation temperature. This study proposed a carbon-negative aragonite production method, demonstrated the possibility of enhanced and controlled aragonite particle production during the CO_2_ mineralization of calcium silicates in the presence of chelating agents.

## Introduction

Given the importance of CaCO_3_ as a filler/extender for the production of paper, paints, plastics, food, pharmaceuticals, and cosmetics^1,2^, much attention has been drawn to the synthesis of high-purity CaCO_3_ particles with controlled crystallographic and morphological characteristics^3,4^. Aragonite is the best filler material among the three CaCO_3_ polymorphs (calcite, aragonite, and vaterite) for paper and plastics industries, and a more useful additive for the biomedical industry^3,5–8^. At present, CaCO_3_ particles are produced using the lime-soda process, the CaCl_2_ (Solvay) process, or carbonation, during which CO_2_ is bubbled into a solution of Ca(OH)_2_. Carbonation is a relatively environmentally friendly and potentially carbon neutral process, as Ca(OH)_2_ is commonly prepared by heating limestone (CaCO_3_), which is accompanied by the release of CO_2_ (CaCO_3_ → CaO + CO_2_; CaO + H_2_O → Ca(OH)_2_; Ca(OH)_2_ + CO_2_ → CaCO_3_ + H_2_O)^4,9^.

The continuous increase in global CO_2_ emissions has drawn much interest to the permanent storage of CO_2_ via its mineralization through reactions with metal (e.g., Ca and Mg) ions extracted from silicate minerals (e.g., CaSiO_3_) or solid wastes (e.g., steelmaking slags and municipal solid waste incinerator ashes)^10–12^. Production of CaCO_3_ particles via CO_2_ mineralization is ideal from the environmental protection perspective since this process could be negative CO_2_ emissions. However, despite the abundance of CO_2_ mineralization strategies (e.g., pH-swing), most of them focus on rock dissolution or CO_2_ mineralization kinetics^13,14^, leaving the control of the crystallographic and morphological characteristics of the as-produced carbonates underexplored. In most cases, calcite is easily precipitated as the most stable phase of CaCO_3_^15,16^, while the precipitation of the metastable aragonite is challenging and usually requires elevated temperatures (e.g., > 75 °C) and strict pH condition (e.g., pH 11), as well as other solution chemical properties control^9,15,17^, or the presence of water-soluble additives (e.g., inorganic metal ions, surfactants, and polymers)^18–20^.

Chelating agents, which effectively bind metals within a wide pH range and offer considerable versatility in industrial and household uses^21,22^, can also promote the extraction of metals from silicate minerals^23,24^, e.g., ethylenediaminetetraacetic acid (EDTA) can significantly enhance Mg extraction from serpentine [Mg_3_Si_2_O_5_(OH)_4_] at pH 7 or 10^24^. Therefore, in CO_2_ mineralization processes, chelating agents may enhance the production of CaCO_3_ by promoting Ca extraction from rocks. However, the generally strong bonding between metal ions and chelating agents may prevent Ca carbonation proceeds in the presence of these agents^25^.

Chelating agents may also influence CaCO_3_ crystallization behavior by changing the surface energy of the solvent-CaCO_3_ crystal interface^18,26,27^. Sun et al. (2014) suggested that the nucleation of the metastable aragonite can be promoted by lowering the interfacial energy^16^. However, different chelating agents may have different effects on interfacial energy. Westin and Rasmuson (2005) reported that the inclusion of chelating agents such as EDTA notably increased the interfacial energy^26^, whereas Townsend et al. (2018) reported that nitrilotriacetamide (NTAA), methylglycine diacetamide (MGDA), and *N,N*-dicarboxymethyl glutamic acid (GLDA) decreased the interfacial energy and thus promoted the precipitation of acicular NaCl crystals^28^. On the other hand, the complexation of Ca ions by chelating agents results in a gradual release of these ions during carbonation and, hence, in a low Ca^2+^/CO_3_^2−^ ratio. Tomiyama and Yasushi (1984) suggested that low Ca^2+^/HCO_3_^2−^ ratios favor the precipitation of aragonite over that of calcite^29^.

**Figure 1:**
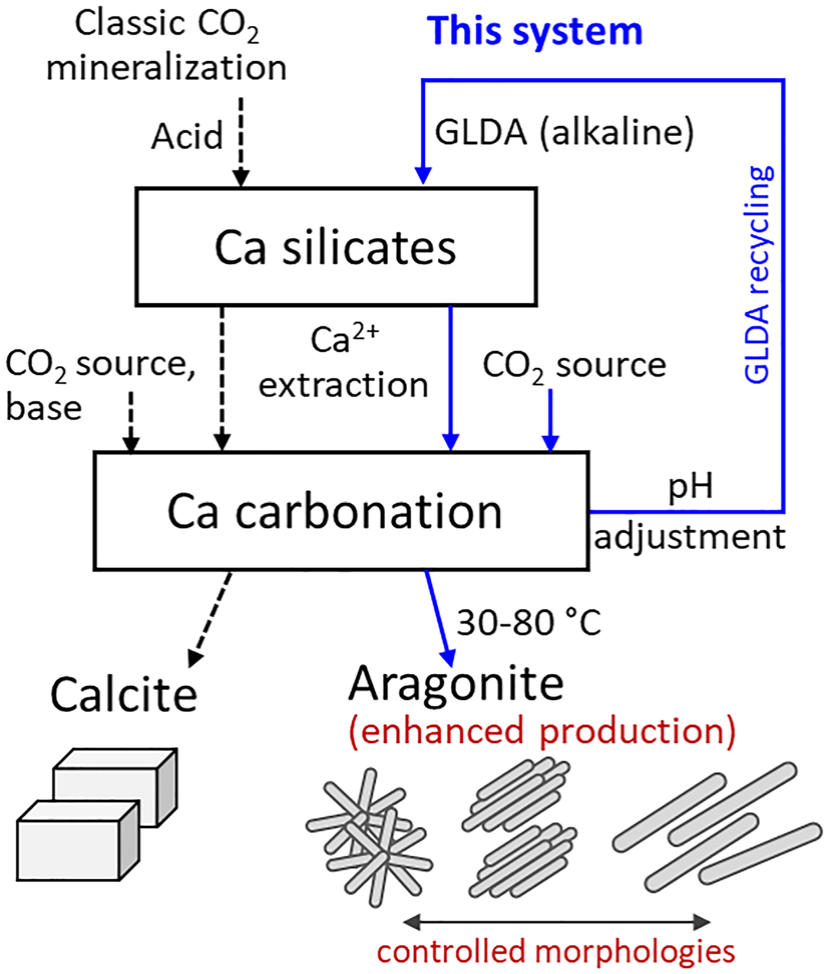
Schematics of GLDA-assisted aragonite production and comparison with a classic CO_2_ mineralization process.

Herein, a chelating agent was used to enhance aragonite production during CO_2_ mineralization, with the system design shown in Fig. 1. GLDA was chosen because of its biodegradability, environmental friendliness, and its good ability to chelate Ca (Ca-GLDA stability constant (log *K*) = 5.9 at an ionic strength of 0.1 M and 25 °C^30^) and lower the surface energy of the solvent-crystal interface for aragonite production^31^. In particular, GLDA was supposed to enhance Ca extraction from calcium silicate, promote the production of aragonite particles with controlled morphologies during the subsequent Ca carbonation, and subsequently be recovered. GLDA concentration, carbonation temperature, and pH were optimized to maximize the aragonite production efficiency as well as the purity and uniformity of aragonite particles.

## Materials and methods

### Materials

**Figure 2:**
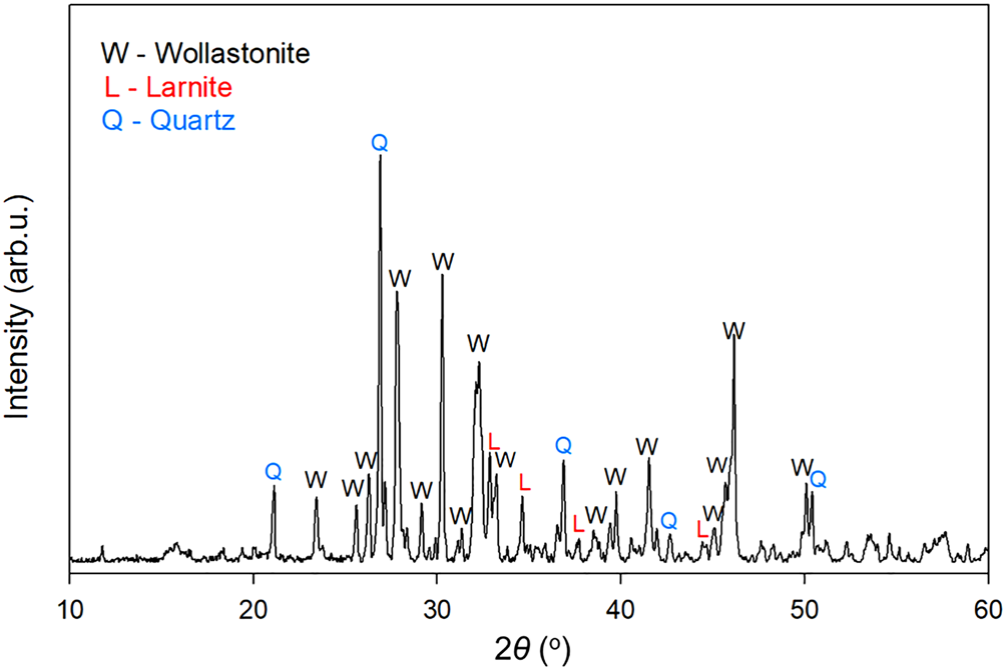
Representative X-ray diffraction (XRD) pattern of the employed calcium silicate.

GLDA tetrasodium salt (GLDA-4Na, C_9_H_9_NNa_4_O_8_) solutions with different concentrations were prepared by mixing the initial GLDA-4Na solution (40 wt% in water, Tokyo Chemical Industry, Japan) with Milli-Q water, and pH was adjusted from an initial value of 13.8–~9.0 through the addition of aqueous HNO_3_ (60–61%, Kanto Chemical, Japan). This weakly alkaline pH was used at the beginning of the Ca extraction process to minimize the cost of pH regulation, as subsequent CaCO_3_ precipitation requires alkaline environments (pH > 8)^32,33^. High-purity commercial calcium silicate powders (Fig. 2, < 30 µm) with a general composition of CaSiO_3_ (Wako Pure Chemical Industries, Japan) were used as a Ca source to represent silicate minerals. Na_2_CO_3_ (>99.0%, Kanto Chemical, Japan), NaHCO_3_ (> 99.0%, Kanto Chemical, Japan), and CO_2_ gas (> 99.995 vol.%, Taiyo Nippon Sanso, Japan) were used as CO_2_ sources to optimize the CaCO_3_ production system.

### Experiment design

All reactions were conducted in beakers. In the first step, calcium silicate powders (1.16 g) were suspended in GLDA solutions (100 mL; 0, 0.01, 0.03, 0.05, 0.10, and 0.30 M) to achieve a calcium silicate concentration of 0.1 M, and the suspensions were continuously stirred (300 rpm) for 20 min at 50 °C. This slightly-higher-than-ambient temperature was selected to accelerate Ca extraction^14^. Samples (~ 1 mL) were withdrawn after 0, 2, 5, 10, and 20 min using a syringe.

The suspensions were filtered through 0.45-µm membranes to remove residual calcium silicate particles, and the obtained transparent GLDA solutions (Ca-rich) were treated with solid Na_2_CO_3_, solid NaHCO_3_, or CO_2_ gas at 30, 50, or 80 °C to trigger Ca carbonation. In the case of using CO_2_ gas for Ca carbonation, high purity CO_2_ gas (> 99.995 vol.%) were injected into the Ca-rich GLDA solutions by bubbling, the flow rate was not recorded in this study. All carbonation experiments were conducted at the atmospheric pressure. Each carbonation experiment was conducted with continuous stirring at 200 rpm for 60 min, with samples (~ 1 mL) withdrawn via a syringe after 20, 40, and 60 min. The total volume of extracted samples was below 10% of the initial GLDA solution volume, and the sampling procedure was therefore assumed to have no major effects on the reactions. After carbonation, the suspensions were filtered through 0.45-µm membranes, and the obtained CaCO_3_ precipitates were analyzed.

### Analytical methods

Solution pH before and after each reaction was measured using a pH meter at room temperature (~ 20 °C). Fluid samples were analyzed by inductively coupled plasma-optical emission spectrometry (ICP-OES; Agilent 5100) to quantify the dissolved components (i.e., Ca and Si). The solid samples collected after Ca carbonation were filtered, washed with Milli-Q water, and dried at 50 °C for > 24 h before measurements. Crystal structures were identified by XRD (Multiflex, Rigaku, Japan) at 40 kV and 15 mA using Cu *K*_α_ radiation, a 2*θ* range of 10°–60°, and a scan step of 0.02°, and the acquired data were analyzed using MDI Jade 6 software^19^. Surface morphologies were characterized by SEM (SU-8000, Hitachi, Japan) coupled with energy-dispersive X-ray spectroscopy (EDS). Crystallite sizes were estimated using MDI Jade 6 software according to the Scherrer equation. Instrumental broadening and crystallinity were not considered, as they were supposed not to influence the crystallite variation trend^9,20^. Particle sizes were determined using SEM.

## Results and discussion

### GLDA-enhanced Ca extraction from calcium silicate

**Figure 3:**
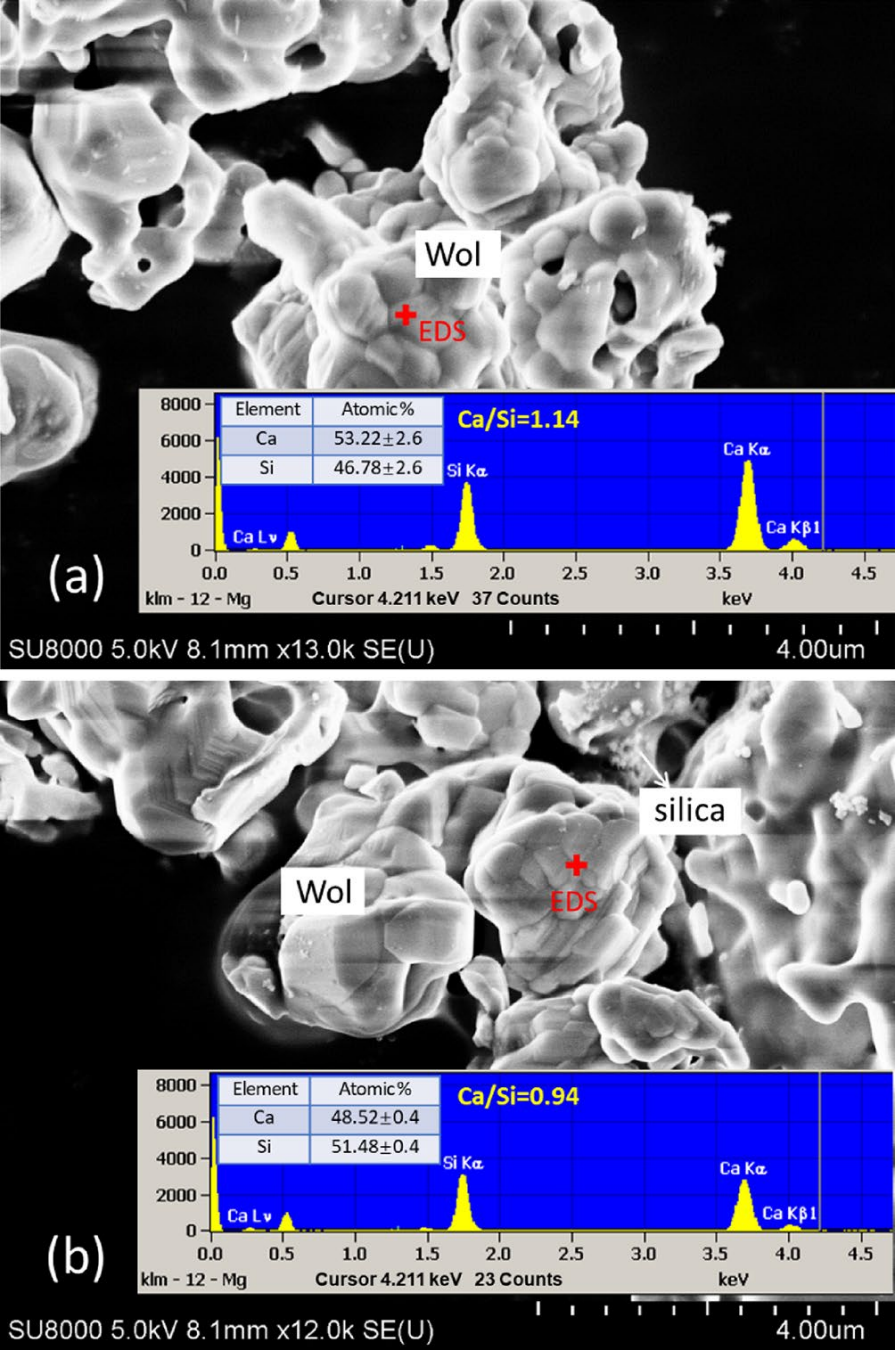
SEM images and EDS results of calcium silicate particles (**a**) before and (**b**) after 20 min treatment with 0.3 M GLDA. Wol: wollastonite.

In all experiments, Ca extraction was almost complete in 20 min. The faster Ca extraction at the beginning may be attributed to the dissolution of smaller calcium silicate particles, as suggested by Gadikota et al. (2014), that silicate particles smaller than 10 µm dissolved much faster than coarser particles^34^. However, according to SEM imaging, extraction had no obvious effect on the appearance of calcium silicate particles (Fig. 3). The difficulty in size observation may be related with both the aggregation effects and the overall decrease in particle size.

**Figure 4:**
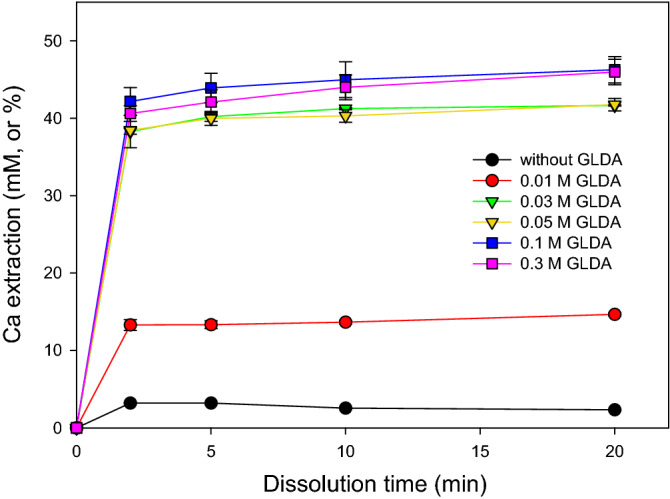
The changes of Ca concentrations (or extraction ratio, %) with dissolution time in the presence of 0−0.3 M GLDA.

The extraction conditions and solution chemistries obtained after 20 min were summarized in Table 1 and Fig. 4. The Ca extraction efficiency of 41.64% reached after 20-min treatment with 0.03 M GLDA was 17.9 times higher than that obtained without GLDA under identical conditions (2.33%). Given that one chelating agent molecule usually binds to one metal ion, the Ca/GLDA molar ratio of 1.39 observed for 0.03 M GLDA implied that almost all GLDA molecules were used for Ca bonding. However, with a further increase in GLDA concentration to 0.3 M, the Ca extraction efficiency did not increase, and the Ca/GLDA molar ratio decreased to 0.15, which suggested that the main factor preventing the improvement of Ca extraction efficiency was the change in CaSiO_3_ particle properties. For instance, an incongruent dissolution of Ca and Si (Ca/Si in solution ≥ 2.06) was observed, i.e., the dissolution of Ca was preferred, while Si preferentially remained on the calcium silicate surface (Table 1). When the GLDA concentration was decreased from 0.03 to 0.01 M, the Ca extraction efficiency dramatically decreased to 14.66% (Table 1), and the Ca/GLDA molar ratio reached 1.47, i.e., extraction was hindered by the insufficient amount of GLDA. Therefore, a GLDA level of 0.03 M was concluded to be optimal for the extraction of Ca from 0.1 M calcium silicate. At this GLDA concentration, the levels of both GLDA-bonded and free Ca ions were maximized.

**Table 1:** Conditions of the calcium silicate-GLDA reaction and fluid chemistries obtained after 20-min extraction.^a^

Exp.	GLDA (M)	pH_0_^b^	pH_d_^b^	Ca extraction (mM, or %)	Si extraction (mM, or %)	Ca/Si mol/mol	Ca/GLDA mol/mol
1	0	9.1	11.7	2.33	0.53	4.46	–
2	0.01	9.0	12.0	14.66	4.21	3.51	1.47
3	0.03	8.9	12.3	41.64	19.63	2.12	1.39
4	0.05	9.0	11.9	41.75	20.26	2.06	0.83
5	0.10	9.0	10.0	46.25	21.89	2.12	0.46
6	0.30	9.0	9.3	45.96	2.84	16.73	0.15

^a^All experiments were conducted with 0.1 M Ca silicate at 50 °C. Each experiment was repeated twice and the average values were shown, the experiments showed good reproducibility within a margin of error of 3%.

^b^“pH_0_” refers to the initial pH, and “pH_d_” refers to the pH after dissolution. Both pH values were measured at room temperature.

The extraction efficiencies of Si were lower than those of Ca, as the Ca/Si molar ratio in the solution was greater or equal to 2.06 in all cases (Table 1). EDS measurement of the surface of calcium silicate particles also revealed a slightly decrease in Ca/Si ratio from 1.14 to 0.94 after 20 min treatment with 0.3 M GLDA (Fig. 3). Nevertheless, more Si was extracted with ≥ 0.03 M GLDA than in its absence, which implies that this chelating agent can suppress the formation of a silica-rich layer to some extent to facilitate the release of Ca from calcium silicate. The formation of silica-rich passivating layers (with thicknesses ranging from several nm to µm) has been considered as one of the major contributors to the suppressed dissolution of metal ions from silicate minerals during CO_2_ mineralization^12,35^.

In all experiments, pH increased after 20-min extraction (Table 1), which was ascribed to silicate dissolution or/and chelation^24,36–38^. Generally, GLDA concentration was negatively correlated with the extent of pH change, which was attributed to the buffering effect of GLDA^4+^ and H-GLDA^3+^. In the case of 0.3 M GLDA, pH increased from 9.0 to 9.3 over 20 min. At this pH, the solubility of SiO_2_ is low, and the released SiO_2_ may be precipitated, which may explain the low concentration of Si in the solution (Table 1, Exp. 6).

### Feasibility of aragonite production in the presence of GLDA

The feasibility of CaCO_3_ production in the presence of GLDA was studied using the extract obtained using 0.03 M GLDA (Exp. 3, Table 1; Ca/GLDA = 1.39) and containing 41.64 mM extracted Ca. Excessive Na_2_CO_3_ (0.05–0.3 M), NaHCO_3_ (0.1–0.3 M), and CO_2_ gas were then added to promote Ca carbonation and aragonite production at an elevated temperature of 80 °C^19^. The Ca carbonation efficiency (carbonated Ca/extracted Ca) was determined by assuming that the decrease in Ca concentration was exclusively caused by Ca carbonation.

**Figure 5:**
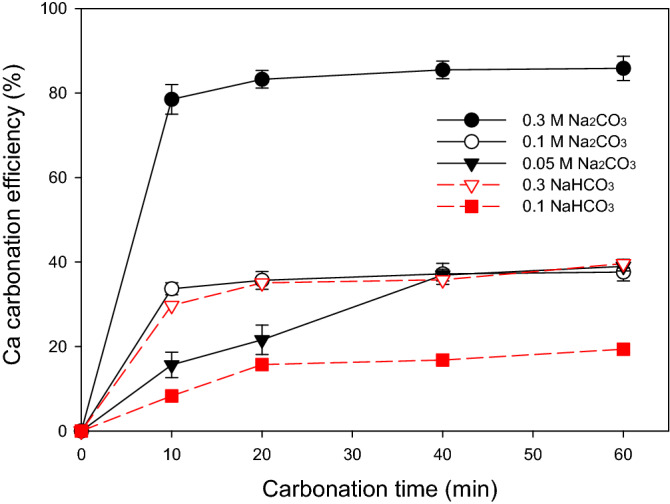
Effects of time and carbonation agent type/concentration on Ca carbonation efficiency. Carbonation experiments with Na_2_CO_3_ addition were repeated twice and the average values were shown.

In most cases, Ca carbonation was fast in the first 20 min but rapidly slowed down in the following period (Fig. 5), as indicated by the rapid decrease in Ca concentration. Carbonation was enhanced by high CO_3_^2−^ concentrations, and the maximal efficiency of 85.84% was achieved using the highest Na_2_CO_3_ level of 0.3 M. At a lower CO_3_^2−^ concentration of 0.1 M, which was still excessive for Ca carbonation, the Ca carbonation efficiency decreased to 37.61% (Fig. 5). Notably, pH was not influenced by Na_2_CO_3_ concentration (Table 2).

**Table 2:** Selected parameters of CaCO_3_ particles obtained under various carbonation conditions. A: aragonite, C: calcite, V: vaterite.

GLDA (M)	Na_2_CO_3_ (M)	*T* (°C)	pH_c_^a^	Phase content (%)^b^			Crystallite size of aragonite (nm)^b^					
							Crystallite orientation					Mean
				A	C	V	111	021	012	200	221	
0.03	0.3	80	12.3	83.2	16.8	–	58.9	58.8	72.9	45.3	58.0	58.8
0.03	0.1	80	12.3	93.3	6.7	–	30.7	29.9	34.4	22.8	32.0	30.0
0.03	0.05	80	12.4	55.1	44.9	–	18.7	17.0	24.4	–	17.3	19.4
NaHCO_3_ (M)												
0.03	0.3	80	9.2	100	–	–	33.8	22.7	27.2	15.3	23.7	24.5
0.03	0.1	80	9.7	97.2	2.8	–	29.9	20.9	22.7	14.3	32.0	22.5

^a^ pH_c_ refers to the pH measured after 60-min carbonation.

^b^ Phase compositions and aragonite crystallite sizes were determined from XRD data using MDI Jade 6.

One the other hand, the addition of 0.1 and 0.3 M NaHCO_3_ reduced the solution pH from 12.3 to 9.7 and 9.2, respectively, and severely suppressed carbonation, and Ca carbonation efficiencies of 19.36 and 39.62%, respectively, were obtained after 60 min (Fig. 5). Direct CO_2_ injection did not trigger Ca carbonation and no obvious precipitation was observed even when the pH was decreased from 12.3 to 7.0 by CO_2_ bubbling, which was attributed to the low concentration of dissolved CO_2_. Thus, to effectively utilize CO_2_ gas for Ca carbonation, the pH should be kept alkaline before a sufficient CO_2_ concentration is reached.

**Figure 6:**
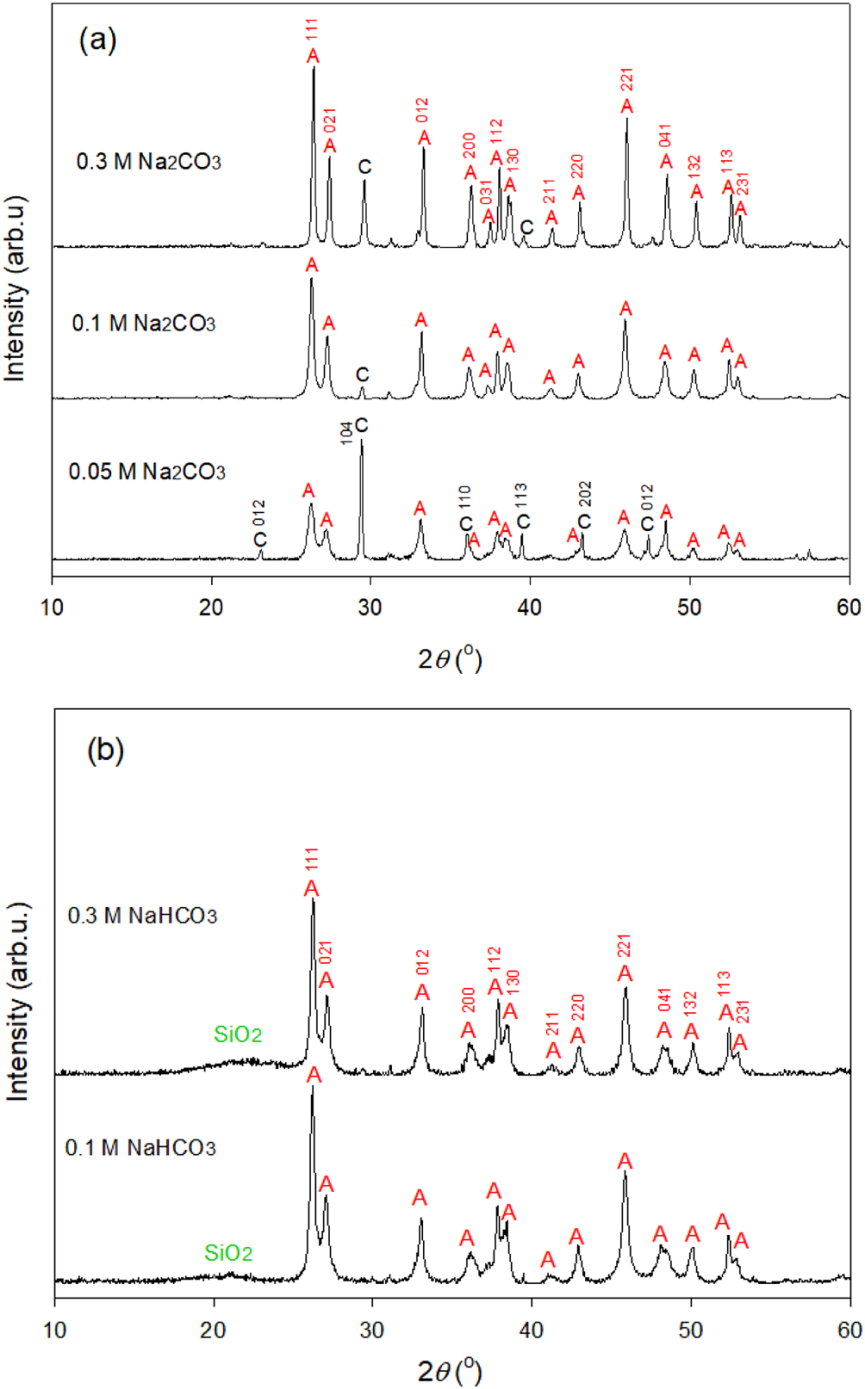
XRD patterns of CaCO_3_ precipitated during carbonation with (**a**) 0.05–0.3 M Na_2_CO_3_ and (**b**) 0.1–0.3 M NaHCO_3_ in 0.03 M GLDA at 80 °C.

**Figure 7:**
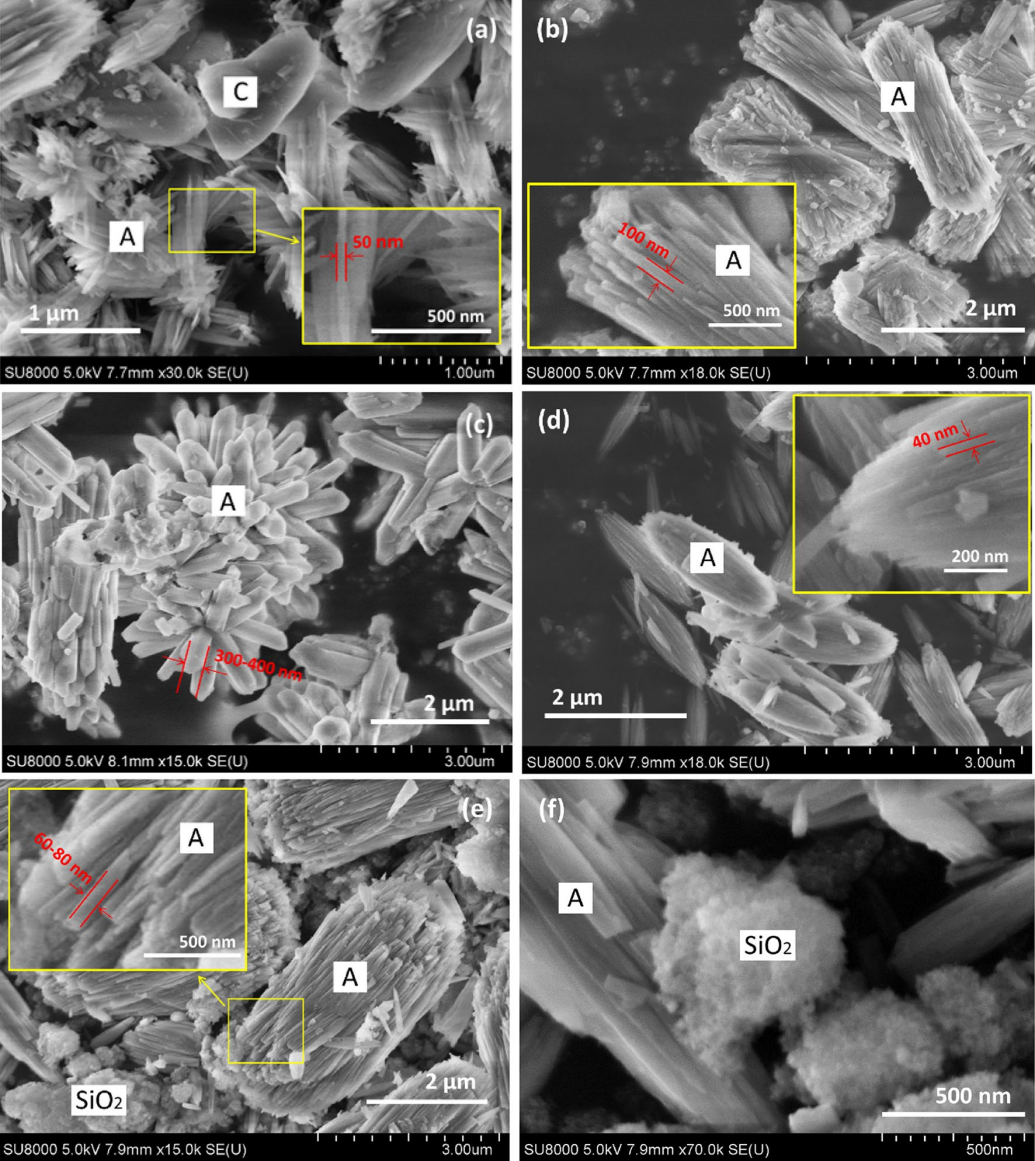
SEM images of CaCO_3_ particles produced using (**a**, **b**) 0.05 M Na_2_CO_3_, (**c**) 0.1 M Na_2_CO_3_; (**d**) 0.3 M Na_2_CO_3_, (**e**) 0.1 M NaHCO_3_, and (**f**) 0.3 M NaHCO_3_ in 0.03 M GLDA (obtained after Exp. 3 in Table 1) at 80 °C.

Higher CO_3_^2−^ concentrations favored aragonite formation, as suggested by the increase in the intensity of aragonite XRD peaks (Fig. 6a). For instance, as the CO_3_^2−^ concentration increased from 0.05 to 0.3 M, the raw intensity of aragonite peak observed at 26.2° increased from 693 to 2215 arb.u. The fast precipitation of CaCO_3_ with the preferred generation of aragonite was consistent with the results of Hirano et al. (2009)^39^. Table 2 lists the mean crystallite sizes determined using 111, 021, 012, 200, and 221 peaks and reveals that high Na_2_CO_3_ concentrations increased crystallite size irrespective of the orientation used for measurement. In particular, as the Na_2_CO_3_ concentration increased from 0.05 to 0.3 M, the mean crystallite size increased from 19.4 to 58.8 nm, and the width of aragonite particles increased from ~ 50 to 300–400 nm (Fig. 7a–c). The fact that aragonite particles were larger than the corresponding crystallites implies that these particles were made up of several different crystallites^40^. Thus, high CO_3_^2−^ concentrations favored aragonite crystallization and nucleation in the presence of GLDA.

The characteristic XRD peaks of calcite became weaker when the concentration of CO_3_^2−^ increased from 0.05 to 0.1 M but gained intensity as the CO_3_^2−^ concentration further increased to 0.3 M (Fig. 6a). The aragonite-to-calcite ratios of each solid sample were semi-determined using MDI Jade 6 under the assumption that all carbonation products were crystalline and detectable by XRD (Table 2). The highest aragonite content of 93.3% was achieved using 0.1 M Na_2_CO_3_, which may be associated with the contradictory effects of CO_3_^2−^ concentration on aragonite (or calcite) formation. A higher CO_3_^2−^ concentration may enhance the production of calcite^3^ but a lower Ca^2+^/CO_3_^2−^ was also reported to suppress calcite formation^19,29^. If this is true, aragonite purity can be controlled by variation of the CO_3_^2−^ concentration. However, this assumption was not verified in the present study.

Aragonite was the dominant CaCO_3_ polymorph obtained at NaHCO_3_ concentrations of 0.1–0.3 M at pH 9.2–9.7 (Fig. 6b). This finding was consistent with the results of Chang and Tai (2010), who found that low pH favors aragonite formation^41^. The produced aragonite particles had relatively small crystallite sizes (mean = 22.5–24.5 nm, Table 2) and dimensions (width = 40–80 nm, Fig. 7d,e). The suppressed aragonite growth may be attributed to low pH, while the increase in NaHCO_3_ concentration had little effect on the enlargement of aragonite crystallites and particles. Based on the above results, low-pH GLDA solutions were expected to induce aragonite production and help to increase aragonite purity; however, an inhibition of aragonite crystallization was observed. At lower pH (e.g., pH ≤ 9.7), amorphous silica precipitation was favored, as evidenced by the broad XRD peak in the range of 15°–25° (Fig. 6b)^42^ and the fine particles observed in the SEM images (Fig. 7), which decreased aragonite purity. Thus, Ca carbonation in the presence of GLDA was concluded to be feasible, and high CO_3_^2−^ concentrations were found to enhance aragonite crystallization and precipitation.

### GLDA-enhanced aragonite production at different temperatures

**Figure 8:**
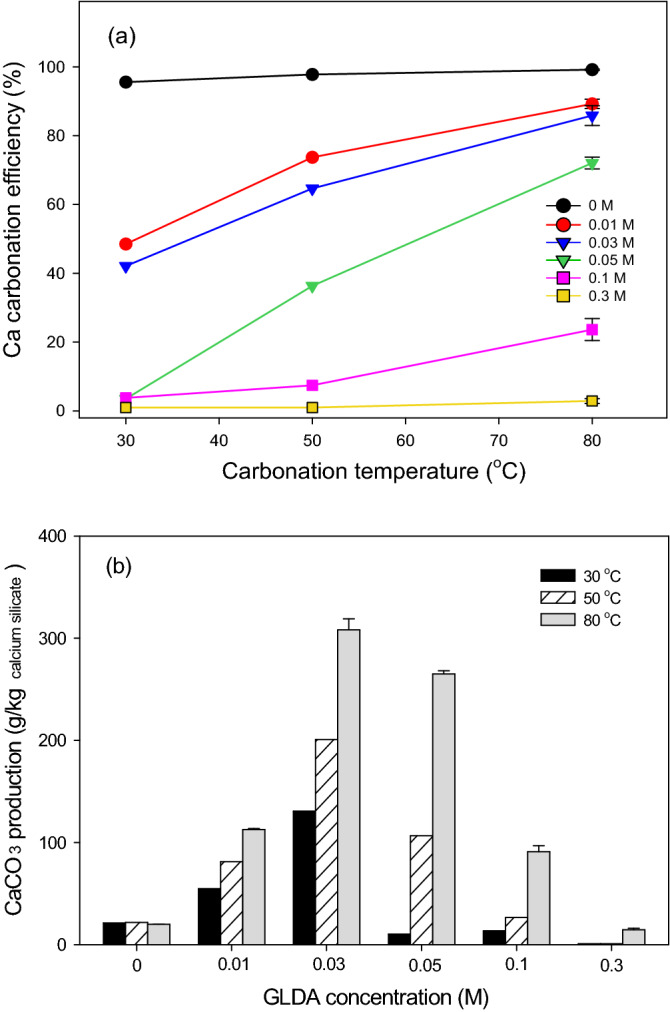
Effects of temperature and GLDA concentration on (**a**) the Ca carbonation efficiency obtained after 60 min using 0.3 M Na_2_CO_3_, and (**b**) CaCO_3_ production efficiency. Carbonation experiments conducted at 80 °C were repeated twice and the average values were shown.

Extracts obtained using GLDA solutions with various concentrations were treated with 0.3 M Na_2_CO_3_ at 30–80 °C. For experiments in which CaCO_3_ precipitation was observed (≤ 0.1 M GLDA), Ca carbonation was fast in the first 20 min but was almost complete in 60 min. Figure 8a presents the Ca carbonation efficiencies obtained after 60 min at 30–80 °C for different GLDA concentrations.

In the absence of GLDA, the carbonation efficiency remained high (> 95%) at all temperatures but significantly increased with temperature at moderate GLDA concentrations of 0.01–0.1 M, reaching 89.24% (0.01 M GLDA) and 85.84% (0.03 M GLDA) at 80 °C. This efficiency was expected to further increase at higher temperatures. Interestingly, Ca carbonation could be triggered at lower temperatures in solutions with lower GLDA concentrations. For instance, at GLDA concentrations of 0–0.03 M, a certain amount of Ca could be carbonated at 30 °C, whereas at a GLDA level of 0.3 M, Ca carbonation was difficult even at 80 °C.

Ca carbonation efficiency was closely related to the final Ca/GLDA molar ratio (i.e., that obtained after 60-min carbonation) at all temperatures. This ratio equaled 0.66 ± 0.03, 0.39 ± 0.04, and 0.19 ± 0.02 after 60-min carbonation at 30, 50, and 80 °C, respectively (the initial values are shown in Table 1), with small differences observed between various GLDA concentrations for each temperature. The decrease in the Ca/GLDA molar ratio with increasing temperature may be due to concomitant changes in parameters such as the Ca-GLDA stability constant, the CaCO_3_ solubility constant^43^, and the carbonate dissociation constant (i.e., *pK*_*a*_ of HCO_3_^−^). Changes in the carbonate dissociation constant may have small effects on CaCO_3_ formation at pH > 12, whereas decreases in the Ca-GLDA stability constant and the CaCO_3_ solubility constant may positively influence Ca carbonation^44^ and thus promote Ca release from Ca-GLDA complexes and the precipitation of CaCO_3_. Thus, CaCO_3_ production during Ca carbonation can possibly be enhanced by increasing the Ca/chelating agent molar ratio during Ca extraction, adjusting the pH or rock dosage, and lowering the Ca-GLDA stability constant.

The CaCO_3_ production per kg of calcium silicate was calculated by combining the results of Ca extraction (Table 1) and Ca carbonation (Fig. 8a), as summarized in Fig. 8b. Compared to that obtained in the absence of GLDA, significantly higher CaCO_3_ production efficiencies were obtained for combinations of 0.01–0.03 M GLDA + carbonation temperature ≥ 30 °C, 0.05 M GLDA + carbonation temperature ≥ 50 °C, and 0.1 M GLDA + carbonation temperature ≥ 80 °C. The optimum GLDA concentration for CaCO_3_ production from 0.1 M calcium silicate was 0.03 M, in which case the highest CaCO_3_ production efficiency of 308 g/kg of calcium silicate was reached in ~ 20 min (35.76% of calcium silicate was carbonated) and was 15.5 times higher than that obtained without GLDA (19.9 g/kg of calcium silicate). Even higher CaCO_3_ production efficiencies were expected at carbonation temperatures above 80 °C in the presence of GLDA. These results suggested that chelating agents can significantly enhance CaCO_3_ production and showed that chelating agent concentration is an important factor that should be controlled at a certain low level, as otherwise, the decrease in the Ca-GLDA stability constant precludes the release of sufficient Ca for carbonation.

### GLDA-controlled CaCO_3_ crystallization behavior

**Figure 9:**
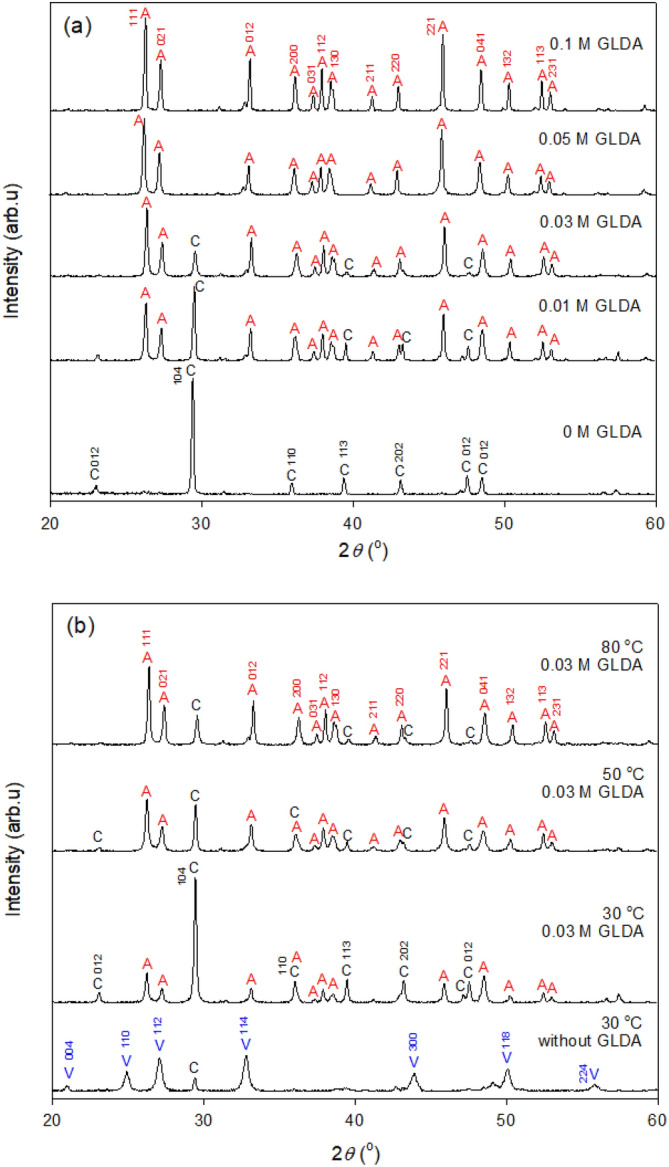
XRD patterns of precipitates formed during Ca carbonation (**a**) at 80 °C, GLDA concentrations of 0–0.1 M, and a Na_2_CO_3_ concentration of 0.3 M and (**b**) at 30–80 °C, a GLDA concentration of 0.03 M and a Na_2_CO_3_ concentration of 0.3 M. A: aragonite, C: calcite, V: vaterite.

In addition to affecting the CaCO_3_ production efficiency, GLDA concentration also influenced the CaCO_3_ precipitation behavior. At a carbonation temperature of 80 °C and high GLDA concentrations in the range of 0–0.1 M, the aragonite XRD peaks were stronger than the calcite peaks (Fig. 9a), which implied that the former polymorph was generated in preference to the latter. Upon the addition of 0.01 M GLDA, the aragonite content increased from 5.1% (without GLDA) to 62.7%, while the calcite content decreased from 94.9 to 37.3% (Table 3). When ≥ 0.05 M GLDA was used at 80 °C, the XRD pattern featured dominant aragonite peaks and no calcite peaks (Fig. 9a), i.e., high-purity aragonite particles were formed (~ 100%, Table 3). Although aragonite production requires a relatively low pH (e.g., 9), the inclusion of GLDA allowed the acceptable pH range to be significantly expanded (i.e., to 9.2–12.3)^17,41^. The increase in GLDA concentration from 0.01 to 0.1 M resulted in an increased aragonite crystallite size for all orientations (from 55.6 to 66.8 nm on average).

**Table 3:** Effects of carbonation conditions on the selected properties of CaCO_3_ particles.

GLDA (M)	Na_2_CO_3_ (M)	*T* (°C)	pH_c_	Phase contents (%)			Crystallite sizes of aragonite (nm)					
							Crystallite orientation					Mean
				A	C	V	111	021	012	200	221	
0	0.3	80	11.59	5.1	94.9	–	–	–	–	–	–	–
0.01	0.3	80	11.74	62.7	37.3	–	49.9	54.6	74.0	40.2	59.3	55.6
0.03	0.3	80	12.28	83.2	16.8	–	58.9	58.8	72.9	45.3	58.0	58.8
0.05	0.3	80	11.89	100.0	–		58.2	61.9	72.1	46.4	59.6	59.6
0.1	0.3	80	10.89	100.0	–	–	72.5	74.3	90.4	65.7	66.8	66.8
0	0.3	30	10.95	–	12.0	88.0	–	–	–	–	–	–
0.03	0.3	30	12.37	33.0	67.0	–	48.0	43.9	43.1	–	57.5	48.1
0.03	0.3	50	12.32	69.2	30.8	–	45.7	40.2	43.9	–	52.4	45.6

A: aragonite, C: calcite, V: vaterite

The inclusion of GLDA extended the temperature range for aragonite production down to 30 °C, while previous studies employed carbonation temperatures of > 75 °C^45,46^. In the case of 0.03 M GLDA, the aragonite content equaled 33.0% even at a low temperature of 30 °C (Table 3) and increased with increasing carbonation temperature, reaching 83.2% at 80 °C. However, in the absence of GLDA, aragonite contents of ≤ 5.1% were obtained at carbonation temperatures of 30–80 °C. Notably, the size of aragonite crystallites increased only at high temperatures of 50–80 °C (Table 3).

**Figure 10:**
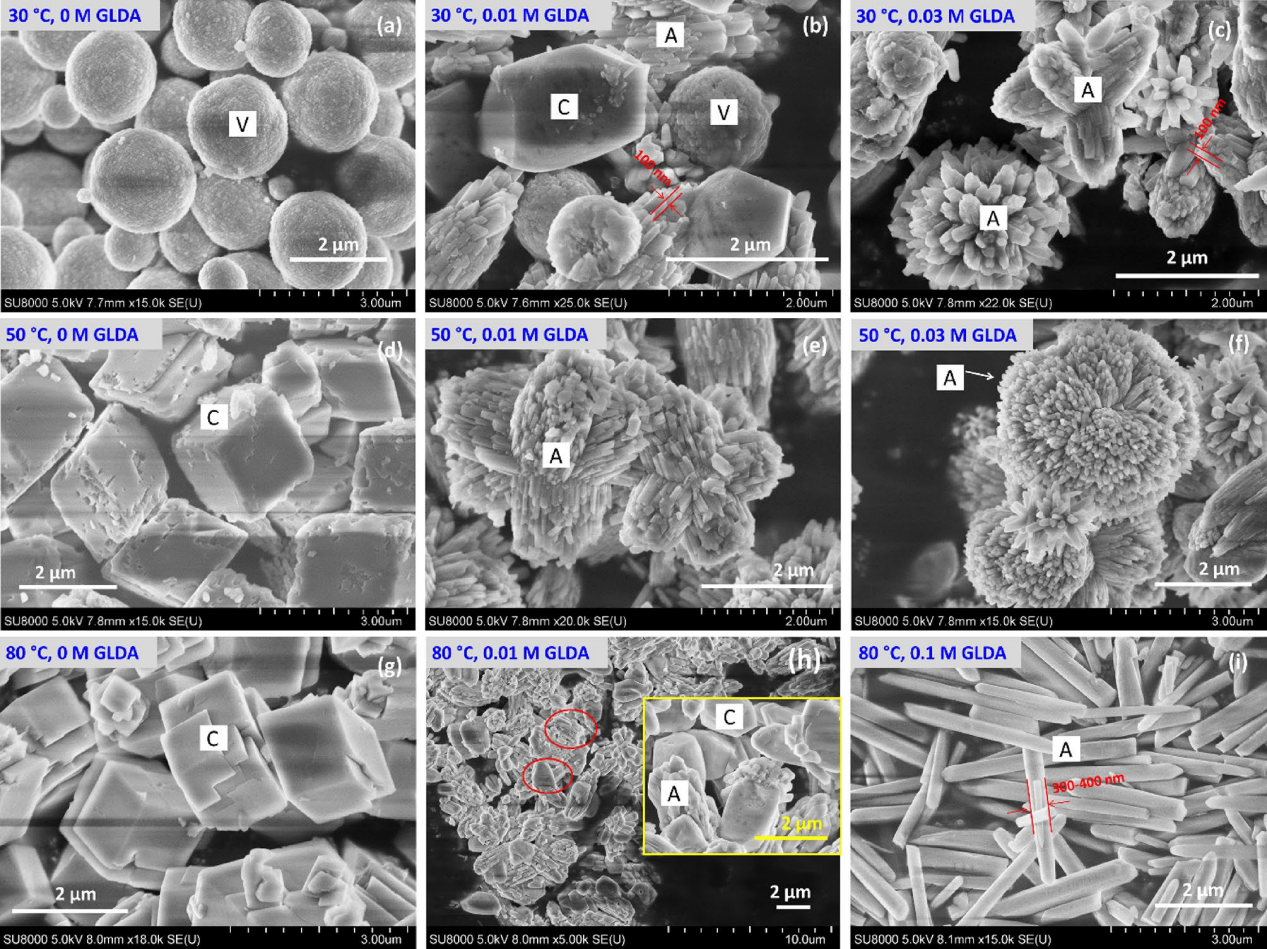
SEM images of CaCO_3_ particles produced in the presence of (**a**,**d**,**g**) 0 M, (**b**,**e**,**h**) 0.01 M, (**c**,**f**) 0.03 M, and (**i**) 0.1 M GLDA at (**a**–**c**) 30 °C, (**d**–**f**) 50 °C, and (**g**–**i**) 80 °C. A: aragonite, C: calcite, V: vaterite.

The morphologies of as-prepared CaCO_3_ are shown in Fig. 10. At a low temperature of 30 °C, spherical vaterite particles were mainly generated in the absence of GLDA (Fig. 10a), in line with the XRD results (Fig. 9b). However, in 0.01 M GLDA, all three CaCO_3_ polymorphs were generated (Fig. 10b). The elongated particles with three end faces on each side of the crystal were regarded as calcite, as suggested by Altay et al. (2007)^18^, whereas bundles of rods were identified as aragonite^20^. Significant variations in aragonite morphology with GLDA concentration were observed. At 30 °C, an increase in GLDA concentration from 0.01 to 0.03 M promoted the clustering of aragonite rod bundles to form bouquet-like aggregates (Fig. 10c). This anisotropic aragonite crystal growth may be associated with the crystallite size increase in 111, 021, and 200 directions.

At carbonation temperatures of 50 and 80 °C, only cubic calcite particles were observed in the absence of GLDA (Fig. 10d, g). However, at a low GLDA level (e.g., 0.01 M), both aragonite and calcite were generated (Fig. 10h). At a constant temperature, the aragonite particle size was not obviously influenced by GLDA concentration in the range of 0.01–0.05 M (Table 1, Fig. 10e, f, h, i). When the GLDA concentration increased to 0.1 M, only needle-like completely separated aragonite particles were observed (Fig. 10i). The formation of these acicular particles may be closely related to the drastic increase in crystallite size in the 012 direction. Moreover, GLDA inhibited the caking of aragonite particles, which may imply a lower surface energy of aragonite^28,47^. In the presence of GLDA, Ca ions were gradually released from Ca-GLDA complexes for carbonation, and the resulting low Ca^2+^/CO_3_^2−^ ratio was considered to induce aragonite formation^29^. Thus, the enhanced formation of the metastable aragonite in the presence of GLDA was ascribed to the surface energy decrease of CaCO_3_ particles and the low concentration of free Ca^2+^ in the solution during Ca carbonation^16^.

Temperature strongly affected the morphology and size of aragonite particles. For instance, spherical particles comprising numerous aragonite rods were formed when the carbonation temperature was increased from 30 to 50 °C in 0.03 M GLDA (Fig. 10i). The high surface area of these particles makes them promising fillers for the paper industry. On the other hand, the average size of aragonite particles increased from ~100 to 300–400 nm as the temperature increased from 30 to 80 °C in 0.03 M GLDA (Fig. 10c, i), in line with the concomitant increase in aragonite crystallite size (Table 3). It should be noted that with increasing GLDA concentration, the aragonite particle size increased by larger factors than the crystallite size, which indicated that high GLDA levels promoted aragonite nucleation. Thus, we concluded that (1) GLDA can enhance aragonite formation and (2) high-purity aragonite particles with various morphologies can be prepared by adjusting the GLDA concentration and temperature.

**Figure 11:**
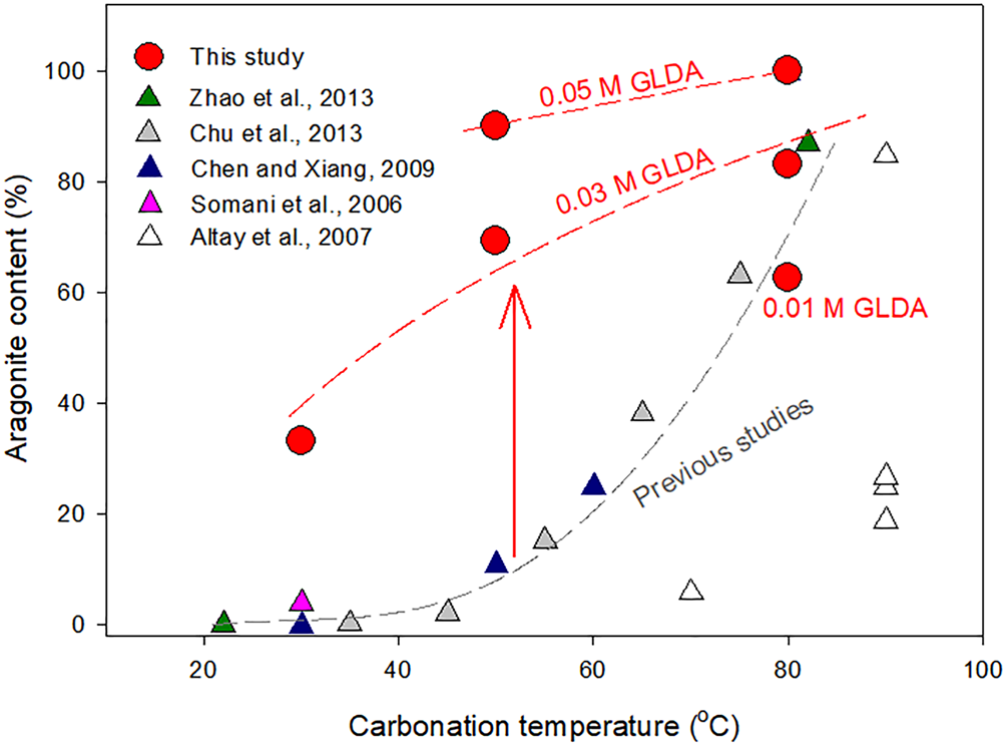
Effects of carbonation temperature on aragonite purity obtained in the present study and other works^3,18,19,48,49^.

The effect of carbonation temperature on the aragonite content of precipitated CaCO_3_ obtained herein and in other studies was compared in Fig. 11. Although various systems have been developed for CaCO_3_ production, aragonite content is generally low at ≤ 70 °C. Herein, the use of 0.03 M GLDA and a carbonation temperature of 50 °C allowed us to increase the aragonite content to > 62%, which is more than seven times that obtained previously at the same temperature^3,18,19,48,49^, and an even higher value of > 90% was achieved using ≥0.05 M GLDA at ≥ 50 °C. The preferential precipitation of aragonite may be associated with surface energy changes and the relatively low concentrations of free Ca^2+^ in the presence of GLDA during Ca carbonation. However, these assumptions were not experimentally verified in the present study. Production of aragonite particles via CO_2_ mineralization of minerals in the presence of GLDA has the advantages of potential negative CO_2_ emissions and relative low cost, the produced aragonite particles also have great application prospects in paper and plastics production owing to their high uniformity and controllable morphology^7^.

Most pH-swing-based CO_2_ mineralization methods require large amounts of acids and bases for rock dissolution and metal carbonation, respectively^33^. However, chelation can occur at both acidic and alkaline pH and thus help to reduce the high cost of pH regulation during CO_2_ mineralization. At the same time, high-purity metastable aragonite with controllable particle morphology can be produced for various industrial purposes, thus providing additional commercial value. The developed system can be improved by injecting CO_2_ gas into the spent GLDA solution to return the pH to the initial value (e.g., 9.0) and thus recover SiO_2_ for GLDA recycling (Fig. 1) and further reduce the operational cost of aragonite production.

## Conclusions

This study investigated the use of chelating agents to enhance and control the formation of CaCO_3_ particles during the CO_2_ mineralization of silicate minerals. The feasibility of the adopted approach was studied using GLDA as a chelating agent and calcium silicate as a representative silicate mineral.

The addition of GLDA accelerated the extraction of Ca from calcium silicate and thus increased the CaCO_3_ production efficiency. In particular, the value of 308 g/kg of calcium silicate obtained under optimal conditions (0.03 M GLDA, pH 9) was 15.5 times higher than that obtained without GLDA. Moreover, GLDA promoted aragonite crystallization and nucleation and controlled its morphology. High-purity and size-/morphology-uniform aragonite particles were obtained in the presence of GLDA even at moderate temperatures (e.g., 50–80 °C) within a wide pH range (e.g., 9.2–12.3). For instance, almost 100% pure aragonite was obtained using ≥0.05 M GLDA at 80 °C, while a purity of only 5.1% was obtained in the absence of GLDA under the same conditions. The aragonite particle morphology could be controlled from bouquet-like to bundles of rods or separated rods, which can be used in various industrial applications.

CaCO_3_ production through chelating agent–assisted CO_2_ mineralization is of both environmental and commercial importance, offering the benefits of negative CO_2_ emission, easy product control, facile operation, and low costs (given that the chelating agent can be recycled). Investigations regarding chelating agent recycling and economic costs will be conducted in a follow-up study.
